# Grain boundary excess volume and defect annealing of copper after high-pressure torsion

**DOI:** 10.1016/j.actamat.2013.12.036

**Published:** 2014-04-15

**Authors:** Bernd Oberdorfer, Daria Setman, Eva-Maria Steyskal, Anton Hohenwarter, Wolfgang Sprengel, Michael Zehetbauer, Reinhard Pippan, Roland Würschum

**Affiliations:** a Institute of Materials Physics, Graz University of Technology, Petersgasse 16, A-8010 Graz, Austria; b Physics of Nanostructured Materials, Faculty of Physics, University of Vienna, A-1090 Vienna, Austria; c Erich Schmid Institute of Materials Science, University of Leoben, A-8700 Leoben, Austria; d Erich Schmid Institute of Materials Science, Austrian Academy of Sciences, A-8700 Leoben, Austria

**Keywords:** Grain boundaries, Grain growth, Dilatometry

## Abstract

The release of excess volume upon recrystallization of ultrafine-grained Cu deformed by high-pressure torsion (HPT) was studied by means of the direct technique of high-precision difference dilatometry in combination with differential scanning calorimetry (DSC) and scanning electron microscopy. From the length change associated with the removal of grain boundaries in the wake of crystallite growth, a structural key quantity of grain boundaries, the grain boundary excess volume or expansion $e_{\mathrm{GB}}=(0.46\pm 0.11)\times 10^{-10}$ m was directly determined. The value is quite similar to that measured by dilatometry for grain boundaries in HPT-deformed Ni. Activation energies for crystallite growth of 0.99±0.11 and 0.96±0.06eV are derived by Kissinger analysis from dilatometry and DSC data, respectively. In contrast to Ni, substantial length change proceeds in Cu at elevated temperatures beyond the regime of dominant crystallite growth. In the light of recent findings from tracer diffusion and permeation experiments, this is associated with the shrinkage of nanovoids at high temperatures.

## 1 Introduction

Processing by severe plastic deformation (SPD) has been established as one of the most promising routes to produce bulk ultrafine or even nanocrystalline materials. These materials made by SPD exhibit exceptional mechanical properties (see e.g. Refs. [1–4]). A comprehensive understanding of these enhanced properties and of the process of grain refinement during severe plastic deformation is currently a major research topic in materials science. In particular, the roles of the various types of defects produced during deformation are widely studied. Furthermore, from a basic materials physics point of view, SPD metals offer the opportunity to study different types of deformation-induced defects and their mutual interaction. It has already been shown that athermally produced excess vacancies are present in high concentrations that are otherwise found only close to the melting temperature [5,6]. A complex defect annealing kinetics is suggested from volume and grain boundary diffusion studies in SPD-processed Cu and Ni [7,8]. During annealing, abundant and highly mobile vacancies may, for example, agglomerate or form a percolating porosity network in combination with triple junctions of grain boundaries. In addition, ultrafine-grained SPD materials give access to basic physical key parameters and processes such as thermally activated grain boundary relaxation prior to grain growth, as well as to the grain boundary excess volume, often also denoted as grain boundary expansion, of relaxed high-angle grain boundaries [9]. For this purpose, the direct experimental method of difference dilatometry is applied in the present work in combination with differential scanning calorimetry (DSC) and scanning electron microscopy (SEM). The subjects of the present study are high-purity samples of the face-centered cubic (fcc) metals Cu and Ni. Novel results obtained from difference dilatometry for copper are compared with results obtained by the other above-mentioned methods, as well as with results previously obtained for pure nickel.

## 2 Experimental

A Cu disk with a purity of 99.995 wt.% was deformed by high-pressure torsion (HPT) at room temperature, with six revolutions being applied at 2.2 GPa (for details see Refs. [10,11]). Samples were cut from this HPT-deformed disk (30 mm in diameter and 7 mm in height) at distances of at least 7.3 mm from the center. This corresponds to a von Mises equivalent strain of ε>23, ensuring a regime, where the deformation is in saturation, i.e. where further deformation will not lead to further grain refinement.

**Fig. 1:**
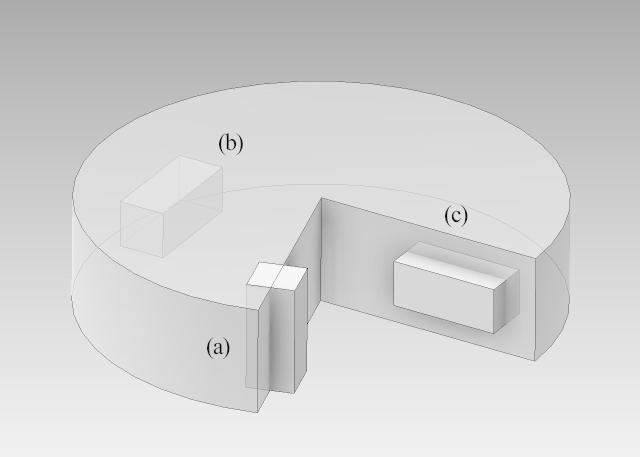
Scheme of an HPT disk (diameter: 30 mm, height: 7 mm), with the dilatometric samples (dimensions: $3\times 3\times 7\;{\text{mm}}^{3}$) denoted as (a) axial, (b) tangential and (c) radial with respect to the direction of the HPT axis.

For the dilatometric measurements, a total of nine prism-shaped specimens with the dimension of $3\times 3\times 7\;{\text{mm}}^{3}$ were prepared. The direction of the length change measurement is defined with regard to the HPT deformation axis (see Fig. 1, axial, tangential and radial directions). Here, seven samples were prepared in the axial direction and one each in the tangential and radial directions.

Experiments were performed with a high-precision, vertical double-dilatometer (Linseis, L75VD500 LT), which allows the simultaneous measurement of two samples under an argon (5 N) gas flow. One of the two samples served as a reference and was made from the same Cu material, which was well annealed and coarse grained (grain size >100μm). The experimental data, plotted as a dilatometric length change curve $\Delta l/l_{0}$, represents the difference signal between the specimen and the reference (so-called difference dilatometry). The length change is directly related to the defect volume via the relation $3\times \Delta l/l_{0}=\Delta V/V_{0}$, assuming an isotropic distribution and annealing of defects [12]. The temperature of the maximum defect release rates can be determined from the minima of the derivative $d(\Delta l/l_{0})/\mathrm{dT}$ of the length change curve with respect to temperature. For the case of a constant linear heating rate, dT/dt, the temperature, *T*, is directly proportional to the time, *t*.

For the DSC measurements, 14 samples, taken from three different positions of the HPT disk, were prepared. Six samples were cut from the disk at a radius of r=9mm at different heights. Four samples were prepared at radii of *r* = 9.3 and *r* = 7.3 mm. The measurements were performed with a Perkin Elmer DSC7 differential calorimeter, which determines the heat release for the annealing processes at different linear heating rates. A subsequent re-run served as the reference measurement and baseline for the analysis (for details see Ref. [13]).

Crystallite sizes were determined by a scanning electron microscope (LEO 1525 field emission scanning electron microscope, with a nominal resolution of 1.5 nm at 20 kV) equipped with a backscattering detector. For correlating the dilatometric length changes with modifications of microstructure, microscopy samples were prepared from the same part of the HPT disk as the dilatometric samples and subsequently annealed under identical conditions in the dilatometer up to predefined temperatures at a heating rate of 5 K/min, followed by rapid cooling to ambient temperature at a rate of about 30 K/min.

The crystallite size was determined from the SEM images by analyzing the area of the grains using the software program ImageJ [14]. A spherical grain shape was assumed for the analysis, and the arithmetic mean value of the diameters was calculated. The number of grains evaluated in each micrograph was about 380 except for the totally recrystallized sample annealed at 673 K, for which about 130 grains were considered. The error in the 1% range is due to uncertainties of assigning contrast features to grain boundaries.

## 3 Results

### 3.1 Correlation between microstructure and free volumes

**Fig. 2:**
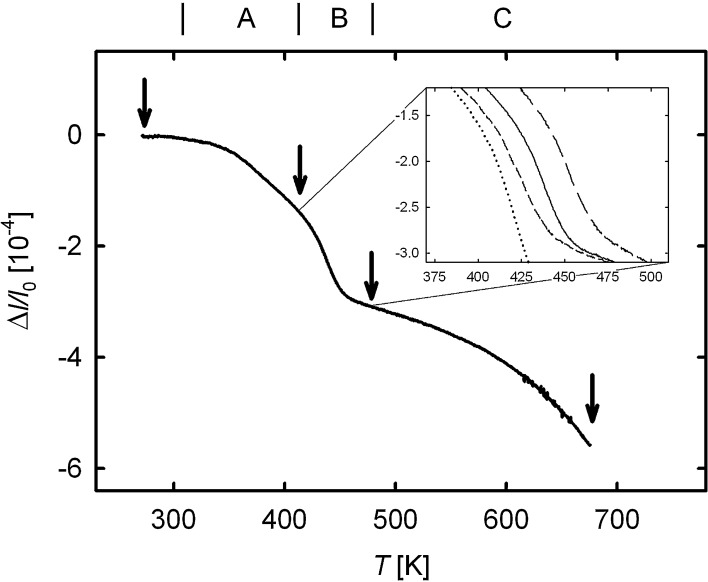
Relative length changes $\Delta \ell /\ell_{0}$ as determined by dilatometry on HPT-deformed Cu with a constant heating rate of 5 K min^-1^ (solid line). The inset shows the shift of the recrystallization process for heating rates of 1.25, 2.5, 5 and 10 K min^-1^ (from left to right). Arrows indicate annealing states for which SEM micrographs were taken (cf. Fig. 3). The letters A, B and C denote the annealing stages discussed in the text.

**Fig. 3:**
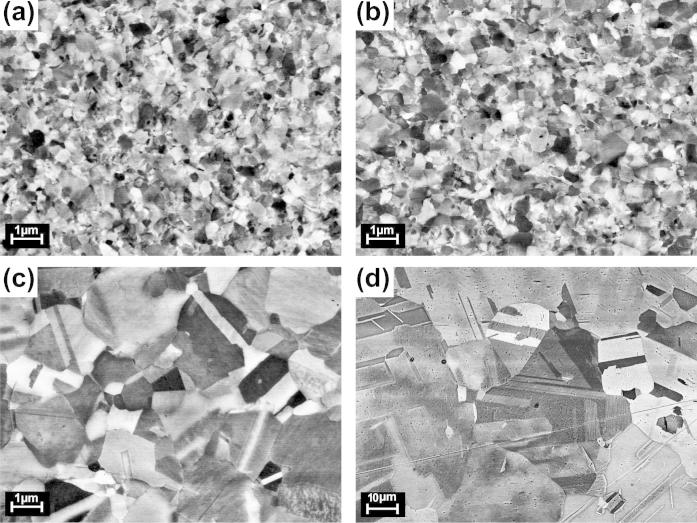
SEM micrographs of HPT-Cu taken at the annealing state indicated in Fig. 2. The magnification is the same in (a)–(c) and 10 times smaller in (d). The plane of the micrograph is oriented parallel to the HPT disk (top view, Fig. 1).

Fig. 2 shows a typical dilatometric length contraction $\Delta l/l_{0}$, indicating the annealing out of defects associated with the release of free volume upon linear heating of the HPT-deformed Cu sample. Three substages, A, B and C, can clearly be discerned. In order to correlate these substages with annealing of specific types of lattice defects, a microstructural characterization by means of scanning electron microscopy was performed at the onset and at the end of each substage, as indicated by arrows in Fig. 2. In stage A, only a minor increase in the crystallite size from 209±4nm (as-deformed, Fig. 3a) to 222±4nm (end of stage A, $T_{a}=413\;\text{K,}\;5\;\text{K}/{\text{min}}^{-1}$, Fig. 3b) is observed. Substantial crystallite growth up to 764±4nm ($T_{a}=468\;\text{K}$, Fig. 3c) takes place in the subsequent distinct annealing stage B. The broad annealing stage C is accompanied by further crystallite growth up to 12.09±0.04μm ($T_{a}=673\;\text{K}$, Fig. 3d). In contrast to HPT-deformed Ni (see below), both the crystallite shape and the dilatometric length change are isotropic. Quite similar dilatometric curves were obtained for the three measuring directions (axial, tangential and radial, Fig. 1).

From the measurements of nine dilatometric samples, a total length change $\Delta l/l_{\text{total}}=(7.02\pm 1.92)\times 10^{-4}$ is deduced that comprises a fraction ${\left(\Delta l/l_{0}\right)}_{\text{Stage A}}=(1.32\pm 0.46)\times 10^{-4}$ for stage A, ${\left(\Delta l/l_{0}\right)}_{\text{Stage B}}=(1.85\pm 0.37)\times 10^{-4}$ for stage B and ${\left(\Delta l/l_{0}\right)}_{\text{Stage C}}=(3.77\pm 1.33)\times 10^{-4}$ for stage C. The results and uncertainties represent the mean value and standard deviation of the nine dilatometric measurements.

Since, during annealing in stage A, the crystallite size and, therefore, the fraction of grain boundaries remain nearly constant, the length contraction in this stage has to be attributed to the annealing out of crystal lattice defects as well as to a relaxation of grain boundaries (see Section 4). In the clearly defined subsequent stage B, the length change is predominantly caused by the removal of relaxed grain boundaries, as evidenced by the increase in the crystallite size by more than a factor of 3 in this narrow temperature regime. This allows one to determine the grain boundary excess volume $e_{\mathrm{GB}}$ from the relative length change ${\left(\Delta l/l_{0}\right)}_{\text{Stage B}}$ in this stage according to

(1) $${\left(\frac{\Delta l}{l_{0}}\right)}_{\text{Stage B}}=e_{\mathrm{GB}}\;\left(\frac{1}{d_{\text{ini}}}-\frac{1}{d_{\text{fin}}}\right)$$

where $d_{\text{ini}}=222\;\text{nm}$ and $d_{\text{fin}}=764\;\text{nm}$ denote the crystallite diameter at the beginning and at the end of stage B, respectively. From the mean value ${\left(\Delta l/l_{0}\right)}_{\text{Stage B}}=(1.85\pm 0.37)\times 10^{-4}$ of nine samples, an apparent grain boundary excess volume $e_{\mathrm{GB}}^{\prime }$ of $(0.58\pm 0.13)\times 10^{-10}\;\text{m}$ can be derived. However, for a more detailed analysis, it should be taken into account that the onset of the annealing processes of stage C already occurs during stage B. Subtracting this contribution by means of extrapolation of stage C to lower temperatures into the stage B regime yields a reduced effective value ${\left(\Delta l/l_{0}\right)}_{\text{Stage B, eff}}=(1.48\pm 0.29)\times 10^{-4}$ associated with the removal of grain boundaries, resulting in a corrected value of the grain boundary excess volume of $e_{\mathrm{GB}}$=$(0.46\pm 0.11)\times 10^{-10}\;\text{m}$.

### 3.2 Kinetics and comparison with DSC

**Fig. 4:**
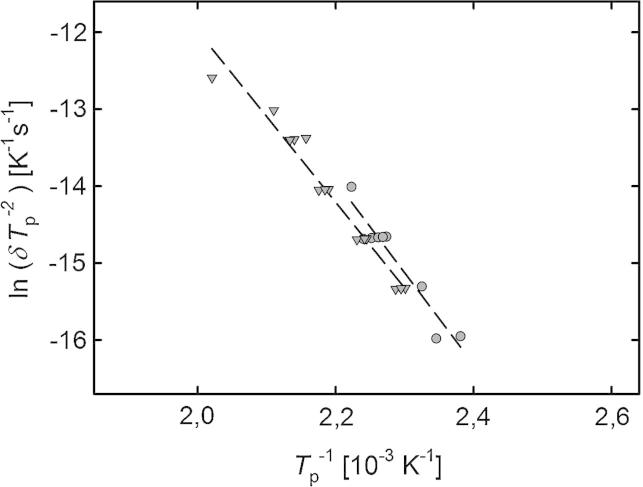
Kissinger plot of the temperatures $T_{p}$ of maximum length change rate (dilatometry: •) or heat release rate (DSC: ▾) measured on HPT-Cu applying different heating rates δ in the range from 1.25 to 50 K min^-1^.

The shift of the dilatometric length change in stage B with heating rate (see the inset in Fig. 2) provides insight into the kinetics of crystallite growth. Analyzing this shift for the nine dilatometric measuring runs according to Kissinger (for details of the method see Ref. [15]) yields an activation energy *Q* of crystallite growth of 0.99±0.11eV (see the Kissinger plot, Fig. 4).

**Fig. 5:**
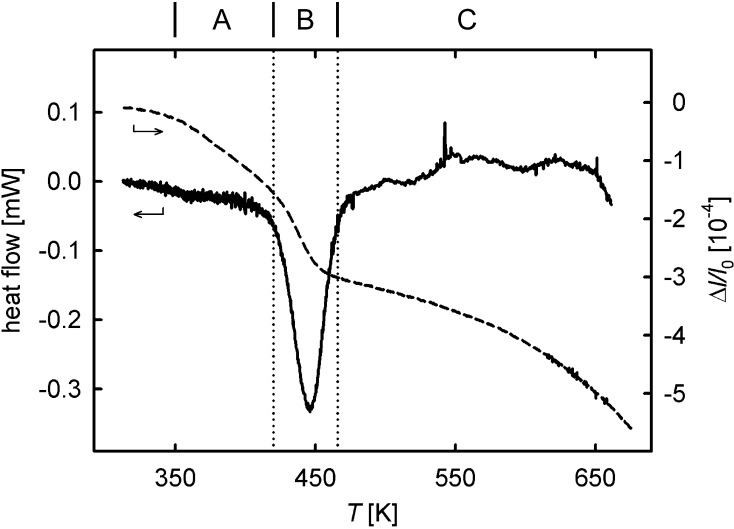
DSC heat flow (—) as well as relative length change (- - -) of HPT-Cu upon annealing up to 673 K at 5 K min^-1^. The annealing stages A, B, and C are indicated.

For the sake of comparison, a series of 14 DSC measurements with different heating rates were taken on samples prepared from the same HPT disk. As shown in Fig. 5, the dilatometric stage B due to crystallite growth is accompanied by a pronounced heat release. Kissinger analysis of the shift of this DSC peak with heating rate yields an activation energy *Q* of 0.96±0.06eV, which is in excellent agreement with the dilatometric measurements (see Fig. 5).

From the heat release upon linear heating through stage B, a mean value of the enthalpy of $\Delta H=-0.92\pm 0.06\;\text{J}\;{\text{g}}^{-1}$ for the exothermic process is deduced from the various measuring runs. Attributing this enthalpy release exclusively to the removal of grain boundaries in stage B, a specific grain boundary energy

(2) $$\gamma =\frac{H\rho }{3\left(d_{\text{ini}}^{-1}-d_{\text{fin}}^{-1}\right)}=0.85\pm 0.08\;\text{J}\;{\text{m}}^{-2}$$

is estimated using the initial and final crystallite diameters of stage B, as given above ($d_{\text{ini}}=222\;\text{nm}$, $d_{\text{fin}}=764\;\text{nm}$), as well as the Cu bulk value of $8.92\;\text{g}\;{\text{cm}}^{-3}$ for the mass density ρ. This value of γ is typical of relaxed grain boundaries in Cu (see Ref. [16] and references therein).

The results for Cu of the present work are summarized in Table 1, together with data previously obtained for Ni [9,15] for comparison.

## 4 Discussion

**Fig. 6:**
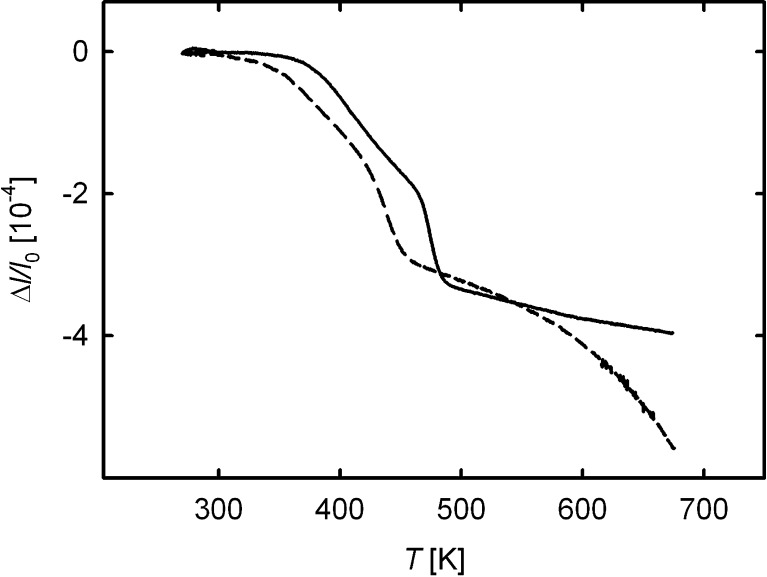
Comparison of dilatometric relative length changes $\Delta \ell /\ell_{0}$ measured on HPT-deformed Cu (dashed line, present work, heating rate $\delta =5\;\text{K}\;{\text{min}}^{-1}$) and on HPT-deformed Ni [15,9] (heating rate $\delta =3\;\text{K}\;{\text{min}}^{-1}$).

For the discussion of the present dilatometric studies of free volumes in HPT-deformed Cu, first of all a comparison with recent results on HPT-deformed Ni [9,15,17] is instructive. The Ni samples were HPT-deformed under identical conditions as in the present case for Cu, and the sample purity (99.99+ wt.%) was also similar. As shown in Fig. 6, the dilatometric change in HPT-Ni exhibits a qualitatively similar three-stage behavior. In particular, a well-defined narrow stage B also occurs in HPT-Ni due the removal of grain boundaries in the wake of pronounced crystallite growth in this stage [9], similar to Cu. However, a number of distinct differences between HPT-Ni and Cu should be noted:

(i) Stage B in Cu is shifted to lower temperatures by about 40 K compared to Ni for similar heating rates.
(ii) The crystallites of HPT-deformed Cu exhibit an isotropic shape, in contrast to HPT-Ni, where a pronounced elongation of the crystallites in the direction tangential to the HPT disk occurs, giving rise to a strong variation in the dilatometric length change with measuring direction, unlike in Cu.
(iii) More than 50% of the total length change in HPT-Cu actually occurs in stage C, whereas for HPT-Ni the regime beyond stage B makes only a minor contribution.
(iv) Stage A, on the other hand, is slightly more pronounced in HPT-Ni (${\left(\Delta \ell /\ell_{0}\right)}_{\text{Stage A}}=1.57\times 10^{-4}$, mean value of 14 samples) than in Cu (${\left(\Delta \ell /\ell_{0}\right)}_{\text{Stage A}}=(1.32\pm 0.46)\times 10^{-4}$).

The shift of the crystal growth-associated stage B towards lower temperatures in Cu compared to Ni (item i; Fig. 6) reflects the different melting temperatures. The same is the case for the activation energies of Q=0.99 and 1.20 eV determined for Cu and Ni, respectively, from Kissinger analysis (Cu, present studies; Ni [15]) and from Johnson–Mehl–Avrami–Kolmogorov analysis (Ni [15]). The value of *Q* = 0.99 eV for Cu is identical to that reported by Číček et al. [18] (1.0 eV) for HPT-Cu. Lower values were reported by Setman et al. [13] (0.48–0.78 eV), Cao et al. [19] (0.8 eV), and by Molodova et al. [20] (0.68 eV), depending on the applied shear strain. A remarkably higher value of 1.68 eV, caused by impurities of oxygen and phosphorus segregating at grain boundaries, was observed by Amouyal et al. [21].

In addition to this issue of kinetics, one should point out the major novel aspect in characterizing SPD materials by means of dilatometry: namely, the access to the absolute concentration of free volumes, and in particular to the grain boundary excess volume $e_{\mathrm{GB}}$, the structural key parameter of grain boundaries. For Cu, a value $(0.46\pm 0.10)\times 10^{-10}\;\text{m}$ was deduced from the present measurements, which is slightly higher than recently reported results for Ni [9]. In the case of Ni with elongated crystallites (item ii), nearly identical values of $e_{\mathrm{GB}}=0.35\times 10^{-10}$ and $0.32\times 10^{-10}\;\text{m}$ were found for the dilatometric measuring directions perpendicular to and parallel to the crystallite elongation, respectively, which confirms the attribution of the length change in stage B to grain boundaries [9]. As any relaxation processes of the SPD-generated grain boundaries should be finished at the elevated temperatures of stage B, these values of the excess volume can be considered as characteristic values for grain boundaries of polycrystalline Cu and Ni in general. It should be noted that the grain boundary excess volume $e_{\mathrm{GB}}$ represents the GB expansion with respect to a perfect crystal lattice and should not be intermixed with the grain boundary width δ, which is usually in the range of 0.5 nm, i.e. much larger than $e_{\mathrm{GB}}$.

Only a few experimental data are available in the literature for grain boundary expansion, primarily for isolated grain boundaries with a distinct orientation relation. From high-resolution transmission electron microscopy, values for Au of $e_{\mathrm{GB}}=(0.04\text{"–"}0.10)\times 10^{-10}\;\text{m}$ (Ref. [22]) or $e_{\mathrm{GB}}=0.12\times 10^{-10}\;\text{m}$ (Ref. [23]) are reported. From measurements of the grain boundary contact angle in an Al tricrystal, a value $e_{\mathrm{GB}}=0.64\times 10^{-10}\;\text{m}$ is reported by Shvindlerman et al. [24] applying a thermodynamic model. For nanocrystalline Pd [25] and Fe [26], values of $e_{\mathrm{GB}}=0.23\times 10^{-10}\;\text{m}$ and $0.19\times 10^{-10}\;\text{m}$ were determined from density measurements and modeling of grain growth kinetics, respectively. A number of computer simulations of grain boundaries [27–30] deal with the issue of grain boundary expansion. Here, however, the choice of the interatomic potentials was found to have a substantial influence on the numerical results [27]. Most recently, grain boundary expansion data have been reported from molecular dynamics simulations on Ni, $e_{\mathrm{GB}}=(0.28\text{"–"}0.42)\times 10^{-10}\;\text{m}$ for random high-angle grain boundaries [30] and $e_{\mathrm{GB}}=(0.39\text{"–"}0.41)\times 10^{-10}\;\text{m}$ (at *T* = 1200 K) for Σ5 grain boundaries [29]. Here, the matching of the data values with the data of the dilatometric studies of Cu and Ni is remarkable.

It is worthwhile mentioning a model proposed by Estrin et al. [31], according to which the annealing out of grain boundary excess volume gives rise to linear grain growth instead of a parabolic behavior. From this point of view, it would be interesting to extend the dilatometry to isothermal measurements and to measure the crystallite size in stage B in more detail, in order to derive experimental information on the correlation between the grain growth kinetics and the free volume release.

The different dilatometric characteristics of Cu and Ni with respect to stage A and, particularly, stage C (items iii and iv) is considered to arise primarily from the different behaviors of lattice vacancies in both fcc metals. Lattice vacancies generated in highly abundant concentrations by HPT deformation become mobile at about 360 K in Ni [32], i.e. in the regime of stage A, whereas in Cu lattice vacancies are already mobile below ambient temperature [33]. Therefore, lattice vacancies in Cu may anneal out or form more stable vacancy agglomerates during deformation. This may explain the reduced amplitude of stage A in Cu compared to Ni.

For a discussion of the substantial release of free volumes ${\left(\Delta l/l_{0}\right)}_{\text{Stage C}}=(3.77\pm 1.33)\times 10^{-4}$ in stage C, one must first note that the removal of remnant grain boundaries in this temperature range contributes to only a minor extent. Taking into account the grain boundary excess free volume derived from stage B, a length change ${\left(\Delta l/l_{0}\right)}_{\text{Stage C, GB}}=0.56\times 10^{-4}$ due to the removal of grain boundaries in stage C is derived from the mean crystallite sizes at the onset and the end of this stage, which corresponds to only 15% of the total length change ${\left(\Delta l/l_{0}\right)}_{\text{Stage C}}$. Thus, the major part of stage C is considered to arise from the shrinkage of nanovoids at high temperatures. Indeed, the shrinkage of nanovoids in Cu by self-diffusion in this temperature range is well documented from early studies of transmission electron microscopy of coarse-grained Cu in which nanovoids were generated by precipitation of quenched-in vacancies (see Bowden and Balluffi [34]). Annealing of vacancy agglomerates in Cu at these elevated temperatures is also deduced from residual resistivity measurements after quenching [33]. Nanovoids in equal-channel angular pressing (ECAP)-prepared Ti and HPT-prepared Cu were detected by small-angle neutron scattering [35] and positron annihilation [18], respectively. Most recently, evidence of percolating porosity in HPT-prepared Cu with a high volume fraction $\Delta V/V_{0}$ in the range of $2\text{"–"}3\times 10^{-3}$ was deduced from radiotracer diffusion and permeation experiments [36]. Assuming isotropic annealing, the dilatometric length change in stage C minus the contribution from removal of grain boundaries corresponds to a change volume $\Delta V/V_{0}=3\times \Delta \ell /\ell_{0}$ in the range of $1\times 10^{-3}$, which is smaller than that from the radiotracer experiments by a factor of 2–3. The different amounts of porosity estimated from the tracer technique and in the present dilatometric annealing experiments therefore indicate that only part of the porosity is annealed out up to the maximum annealing temperature of 673 K – especially since the percolating porosity detected by the tracer method may be considered as the lower limit of the total porosity. Indeed, the dilatometric annealing curve shows that the absolute value of the derivative $d/\mathrm{dT}(\Delta V/V_{0})=3\times \Delta l/l_{0}$ is still increasing at the maximum annealing temperature, i.e. the maximum reaction rate of stage C is not yet attained (Fig. 2). Dilatometry experiments up to higher temperatures may clarify to what extent porosity can be further removed by annealing.

The much less pronounced length change in stage C for HPT-deformed Ni (cf. Fig. 6) may indicate a substantially reduced amount of porosity or at least a higher thermal stability of such porosity for Ni compared to Cu. Tracer diffusion and permeation data for ECAP-prepared metals reveal a higher receptivity for percolating porosity in the case of Cu [37] compared to Ni [8]. This may be related to the aforementioned different vacancy characteristics, which in the case of Cu may favor the formation of stable vacancy agglomerates during deformation.

In conclusion, the direct and specific method of high-precision dilatometry has proven to be a powerful tool for the study of free volume in bulk nanocrystalline metals. In addition to issues of defect kinetics, the absolute value of free volumes, such as the grain boundary excess volume, can be measured directly.

## Figures and Tables

**Table 1:** Results for HPT-deformed Cu (present work) and Ni (previous work [9,15]) of grain size, $d_{\text{ini}}$, at the onset of crystallite growth; ΔH, heat release associated with grain boundaries; $Q_{\text{DSC}}$ and $Q_{\text{DIL}}$, activation energy of crystallite growth determined from calorimetry and dilatometry, respectively; $e_{\text{GB}}$, grain boundary expansion; γ, specific grain boundary energy. Where two values in the case of Ni are given, they refer to the long grain axis perpendicular (⊥) and parallel (‖) to the dilatometric measuring direction.

	Purity	$d_{\text{ini}}$ ($10^{-9}\;\text{m}$)	$\Delta H\;(\text{J}\;{\text{g}}^{-1})$	$Q_{\text{DSC}}$ (eV)	$Q_{\text{DIL}}$ (eV)	$e_{\mathrm{GB}}$ ($10^{-10}\;\text{m}$)	$\gamma \;(\text{J}\;{\text{m}}^{-2})$
Cu	99.995	222±4	0.92±0.06	0.96±0.06	0.99±0.11	0.46±0.11	0.85±0.08
Ni	99.99	175⊥[9], 298‖[9]	–	–	1.20 [15]	0.35⊥[9], 0.32‖[9]	–
